# Recent Advances of Conducting Polymers and Their Composites for Electrochemical Biosensing Applications

**DOI:** 10.3390/jfb11040071

**Published:** 2020-09-25

**Authors:** John H. T. Luong, Tarun Narayan, Shipra Solanki, Bansi D. Malhotra

**Affiliations:** 1 School of Chemistry and the Analytical and Biological Chemistry Research Facility (ABCRF), University College Cork, College Road, T12 YN60 Cork, Ireland; 2 Department of Biotechnology, Delhi Technological University, Delhi 110042, India; narayantarun41@gmail.com (T.N.); shiprasolanki.y29@gmail.com (S.S.); bansi.malhotra@gmail.com (B.D.M.); 3 Applied Chemistry Department, Delhi Technological University, Delhi 110042, India

**Keywords:** biosensor, conducting polymer, enzymeless, electropolymerization, polypyrrole, polyaniline, nanomaterials, glucose

## Abstract

Conducting polymers (CPs) have been at the center of research owing to their metal-like electrochemical properties and polymer-like dispersion nature. CPs and their composites serve as ideal functional materials for diversified biomedical applications like drug delivery, tissue engineering, and diagnostics. There have also been numerous biosensing platforms based on polyaniline (PANI), polypyrrole (PPY), polythiophene (PTP), and their composites. Based on their unique properties and extensive use in biosensing matrices, updated information on novel CPs and their role is appealing. This review focuses on the properties and performance of biosensing matrices based on CPs reported in the last three years. The salient features of CPs like PANI, PPY, PTP, and their composites with nanoparticles, carbon materials, etc. are outlined along with respective examples. A description of mediator conjugated biosensor designs and enzymeless CPs based glucose sensing has also been included. The future research trends with required improvements to improve the analytical performance of CP-biosensing devices have also been addressed.

## 1. Introduction

Electroanalysis is commonly performed on a flat electrode of noble metals (gold, platinum, palladium, etc.), carbon-based materials (glassy carbon, highly ordered pyrolytic graphite, boron-doped diamond, etc.), indium tin oxide (ITO), screen printed electrode (SPE), etc. For enhanced sensitivity, the electrode surface is modified with metal nanoparticles, carbon nanotubes, carbon monoliths, quantum dots, graphene/its derivatives, and conducting polymers (CPs). A review of the use of advanced nanomaterials in biosensor construction is available elsewhere [1]. The selective signal response is based on a specific biorecognition element, which is confined to the sensing area of the electrode by physical adsorption or covalent bonding. In this context, CPs with excellent electrical conductivity, high electronic affinity, and low ionization potential play several key roles in biosensor performance. They are easily polymerized on the electrode surface by a noble salt or a dopant to augment conductivity, catalytic activity, and high surface area. High conductivity is always a desired feature to minimize the interface resistance between the electrolytes, resulting in high signal response. In a broad sense, CPs encompass π-conjugated polymers and polymers whose backbone has alternating single and double (or triple) covalent bonds. Since their discovery, CPs have been extensively used in the biomedical field for biosensor fabrication and tissue engineering. The non-conducting polymers can be used to give mechanical support to the sensor membranes, but CPs improve the sensitivity and stability of the sensing devices along with providing strength. Another property that makes CPs ideal matrices for biosensors is their excellent biocompatibility; CPs have shown extremely stable immobilization of biomolecules on their surface with complete retention of their activity. The most common methods used are cross-linking, covalent binding, physical entrapment, and adsorption [2].

Polymers with amino groups (-NH_2_) and carboxyl groups (-COOH) are easily bioconjugated with the required biorecognition molecules such as enzymes, antibodies, and DNA. Thiolated biomolecules, mostly DNA, self-assemble on conducting polymers decorated with gold or silver nanoparticles. Conducting polymers can be incorporated with hydrogels swollen with water to form conducting porous polymer hydrogels with several desired features for biosensing platforms [3]. Conducting porous polymer hydrogels exhibit ultra-high conductivity, high mechanical properties, hierarchical structure, and swelling nature. High porosity facilitates the diffusion of the analyte and its product(s) in and out of the sensing electrode. Hydrogels swell with water, are biocompatible and serve as an excellent milieu to accommodate and prolong the activity of fragile recognition molecules.

Among four classical conducting polymers, polypyrrole (PPY) and its derivatives have been used extensively in biosensor construction. They are biocompatible and protect electrodes from fouling and rarely cause any significant disturbance of the working environment. In some cases, they form perm-selective films to exclude endogenous electrochemically active interferents. Polyaniline (PANI), another common conducting polymer, loses its conductivity at pH levels above 4, so it is commonly used with another conducting polymer, nanoparticles, or carbon nanomaterials, mainly carbon nanotubes (CNT) and graphene. 

Electrochemical polymerization (EP) has three different steps: (i) solubilization of oligomers in the diffusion layer after monomer oxidation, (ii) their deposition by nucleation followed by growth, and (iii) chain propagation by solid-state polymerization [4]. However, a general mechanism for each polymer cannot be easily established as the process is governed by operating parameters, except for the initial oxidation step [4,5]. The properties of electropolymerized films are dependent upon electrode materials, dopants, electrolyte, monomers, pH, and electrochemical deposition methods. Comprehension of nucleation and growth kinetics allows for the tailoring of polymer properties like density, morphology, and crystallinity, according to requirement. CPs with carboxyl or amino groups serve as immobilization matrices for covalent attachment of recognition molecules [6,7,8,9]. Some polymers like polyphenanthroline facilitate the direct electron transfer (DET) from the active site of biomolecules and the electrode [10]. In some cases, a composite of two polymers, PPY and PANI, are used together to anchor the enzyme on the electrode [11].

This review paper provides an updated overview of CPs based biosensing platforms, based on publications during 2018 to 2020. Literature searches frequently encounter several claims for the choice of electrochemical biosensors and immunosensors: simplicity, high sensitivity, robustness, mass production, miniaturization, multiplexing, and portability. A critical assessment of their analytical performances will be addressed, whether CPs can provide an attractive paradigm in terms of preparations and applications. Except for the non-enzymatic detection of glucose, the use of CPs without a biorecognition molecule to form a chemosensor is excluded in this review regardless of whether the target analyte is a biomolecule. To facilitate reading, biosensing platforms based on conducting polymers, polymer composites, and direct electron transfer are discussed separately. The analytical performances of such biosensors are also tabulated in three different tables for comparison. 

## 2. Electrochemical Polymerization

Conducting monomers can be polymerized by chemical or electrochemical methods. The former produces powders in bulk and is suitable for mass production while the latter results in thin films on the electrode surface with controlled thickness and characteristics. In EP, a solvent consisting of the desired monomer and an anionic doping salt is subjected to an anodic potential. The monomer is oxidized to form the radical cation, which reacts with other neighboring monomers leading to the formation of oligomeric products and eventually polymer deposition at the anode. Both the selected solvent and the doping salt must be stable at the oxidation potential required for EP. The choice of the solvent depends upon the target monomer, e.g., pyrrole is water-soluble, but thiophene is not. Acetonitrile and propylene carbonate are commonly used as the solvent due to their high relative permittivities and permit the use of very large potential windows. EP is commonly performed by cyclic voltammetry (CV), potentiostatic/galvanostatic polarization. The cyclic potential sweep procedure is simple but time-consuming due to dead time in potential regions where no polymerization occurs.

*Polypyrrole* (*PPY*): *Synthesis and Mechanisms*. Pyrrole (PY, MW 67.09, C_4_H_5_N, pK_a_ = 4) is water-soluble (45 mg/mL, 0.67 M at 25 °C) [12] and colorless when fresh. It is also soluble in alcohol, ether, dilute acids, and in most organic chemicals [13]. Its corresponding polymer (PPY) can be easily prepared by chemical oxidative polymerization. Thus, the simple casting of PPY by evaporation of a PPY solution is simple, but the resulting film is not compact or uniform. It is also challenging to control the exact amount of PPY on the active electrode area and the film thickness. Thus, simple casting is excluded for the preparation of isolated microelectrodes and their array. In contrast, EP creates a uniform film on only electroactive surfaces with high reproducibility regardless of their dimensions. PPY is widely used in biosensor construction as the film offers several desired features and PY is commercially available. The classical CV technique offers qualitative information on the redox processes in the polymerization initially and is useful to characterize the polymer films. Potentiostatic techniques can be conducted at a constant potential or a constant current mode to provide more quantitative information to elucidate the nucleation mechanism and macroscopic growth.

**Scheme 1:**
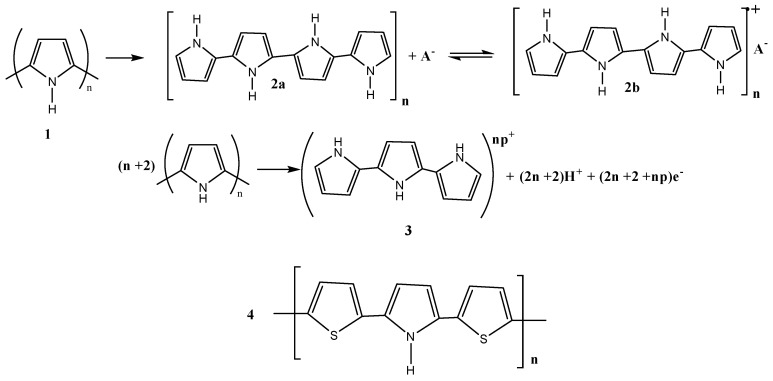
(**1**) Simple structural representation of polypyrrole (PPY); (**2**–**3**) overall reaction of polymerization and doping, and (**4**) poly(2,5-di-(-2-thienyl)-pyrrole.

Among several suggested mechanisms for the formation of PPY, the mechanism proposed by Diaz et al. [14] is frequently encountered in the literature. This mechanism deciphers several successive reactions: radical cation formation, radical coupling, and deprotonation (Scheme 1). It is challenging to probe the propagation and termination steps during EP as intermediate high oligomers are insoluble. The film thickness and morphology deserve a brief discussion here because these important parameters control the diffusion of the target analyte and its corresponding product(s) in and out of the film layer. A supporting electrolyte containing potassium iodide (KI) yields a relatively thick (~0.6 µm) and highly conductive film on a glassy carbon electrode (GCE), whereas KF produces a very thin film (<0.0001 µm) with negligible conductivity [15]. The film thickness is ~0.03–0.04 µm when either KClO_4_, KCl, KBr, or KNO_3_ is included in the electrolyte, compared to <0.01 µm with K_2_SO_4_. EP is performed by CV with 40 cycles from −0.3 to 0.9 V vs. Ag/AgCl, at 0.1 V/s in 0.1 M PY with 0.1 M of the above salts. The film thickness only increases very slowly after the second cycle of CV. The film thickness is also governed by different electrochemical methods and their associated operating parameters. As an example, the PPY film thickness obtained from electrosynthesis at a constant potential of 0.90 V vs. Ag/AgCl, at 20 s and 300 s is about ~2 µm and >25 µm in the electrolyte consisting of 0.1 M PY and 0.1 M potassium iodide [15]. Except for the use of KF, other salts at a concentration of 0.1 M provide a film with thickness ranging from above 5 µm to ~8 µm. In situ atomic force microscopy (AFM) is a great tool to probe the morphology and thickness of the film. For thicknesses below 1 µm, the film exhibits a globular-shaped structure and is independent of the dopant used. Thicker chloride-doped and perchlorate-doped films show cauliflower’s structures [16].

PPY films prepared at lower temperatures have poorer adhesion with an uneven appearance as compared to those prepared at higher temperatures as highly conductive PPY films (500 S/cm) can be prepared at 10 °C [17]. However, if the recognition biomolecule is entrapped by the PPY film in a one-step reaction, the experiment must be conducted at a moderate temperature. The PPY stability in aqueous solution depends on pH as it undergoes protonation in strong acids (pKa of 2–4) and deprotonation in a basic solution (pK_a_ of 9–11). The conductivity decreases from 10 S/cm to 0.1 S/cm when the pH increases from below 7 to pH 11. In most biosensor applications, the recognition biomolecules are compatible at physiological pH. The effect of water on the properties of PPY is another important point. Water exhibits stronger basicity than PY and captures the H^+^ generated during the EP to prevent the formation of the PY trimer [18]. Solvents like methanol, ethanol, and tetrahydrofuran, are more basic as compared to PY and are not capable of deprotonating the intermediate radical cations. Thus, their use is as effective as water to avoid the passivation of the electrode by preventing the plausible formation of the PY trimer. Additionally noteworthy, is the over oxidation of PY, which results in a non-conductive film. For repeated analyses, the PPY film must be firmly attached to the electrode surface, therefore, the deposition of a conducting polymer must be carefully performed and optimized accordingly.

Among several commercial PY derivatives, 4-(3-pyrrolyl)butyric acid and 3-(1*H*-pyrrol-1-yl)-1-propanamine are useful for bioconjugation with the NH_2_ or COOH groups of recognition biomolecules via -NH_2_ bonding, glutaraldehyde crosslinking or carbodiimide covalent bonding between the -NH_2_ and -COOH groups [19]. An electropolymerized pyrrolepropylic acid (PPA) film with a high density of -COOH groups is used to covalently attached protein probes, leading to significantly improved detection sensitivity compared with conventional entrapment methods [9]. Poly(*N*-methyl pyrrole), synthesized from *N*-methyl pyrrole, exhibits a conductivity three orders of magnitude lower than that of PPY [14]. Poly(2,5-di-(-2-thienyl)-pyrrole can be doped with para-toluene sulfonate with electrical conductivities between 10^−8^ and 10^−1^ S/cm [20].

**Scheme 2:**
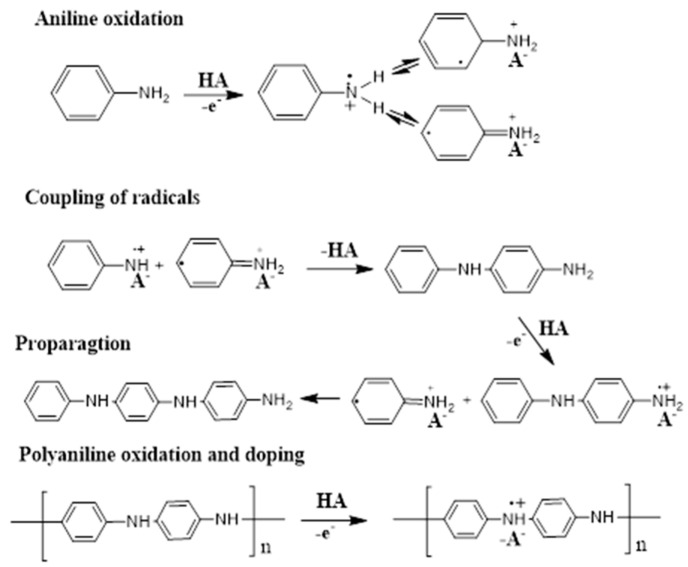
Synthesis steps of polyaniline (PANI) and its resonance structures.

*Polyaniline (PANI): Synthesis and Mechanims*. Like PPY, PANI is prepared by chemical or electrochemical oxidative polymerization of aniline (C_6_H_5_NH_2_, MW = 93.13, solubility in water = 36 g/L at 20 °C). Other techniques involve photochemically initiated polymerization [21,22], and enzyme-catalyzed polymerization [23,24]. EP of aniline is performed in an acidic electrolyte (pH 1–3) as short conjugation oligomeric materials with different properties are formed at higher pH [25]. The first anodic oxidation is rate-determining and irreversible to produce cation radicals [26]. The formation of radicals is confirmed by introducing resorcinol, hydroquinone, benzoquinone, etc. to retard and even terminate the reaction [27]. The subsequent steps are highly dependent upon the reaction conditions if two anilinium radicals at the electrode surface form a dimer (Scheme 2). The stable radical cations react to form low molecular weight products that are soluble with aniline, whereas the unstable cation radicals react very fast with anions or solvent molecules to form undesirable side products [28]. The chain propagation step involves the simultaneous oxidation of the dimer and aniline on the anode followed by the coupling of oligomer radical cations with anilinium radical cation. Finally, anion from the dopant adds to the polymer backbone to form the conducting polymer (Scheme 2).

The application of polyaniline in biochemical systems might be limited as this polymer losses its activity at pH above 4 [29,30]. The deposition of aniline (0.1–1.0 M) is commonly performed under sulfuric acid [31] or hydrochloric acid [32]. Aniline can also be electropolymerized in aqueous electrolytes, acetonitrile, and ionic liquids [11,25]. The introduction of “so-called” pH functional groups such as sulfo, carboxyl, or hydroxyl has been attempted [30]. Aniline can be co-polymerized with *o*-aminobenzoic acid, *m*-aminobenzoic acid, or *m*-aminobenzensulfonic acid to gain redox activity at high pH [25]. PANI can also be used with CNTs [33] and other nanomaterials for electrode modification as discussed later. PANI is the first commercial CP, and some sulfonated aniline derivatives are also commercially available. This polymer serves well as a matrix for the immobilization of biomolecules and might function as a mediator for electron transfer in some enzymatic reactions. PANI can be synthesized in the form of varying nanostructures like nanospheres, nanowires, nanorods, and nanotubes. Besides glucose, PANI has been employed in various chemosensing and biosensing platforms, e.g., in the detection of cancer biomarkers, bacteria, infectious diseases, heavy metals, drugs, phenolics, pesticides, sulfites, acrylamides, etc. [34]. Like other conducting and non-conducting polymers, composites of PANI with other CPs, metallic nanoparticles, graphene, CNTs, etc. have increased surface area, conductivity, and stability. Such desired features play an important role in the development of analytical platforms for a variety of analytes valuable in the food industry, medicine, and environmental monitoring.

**Scheme 3:**
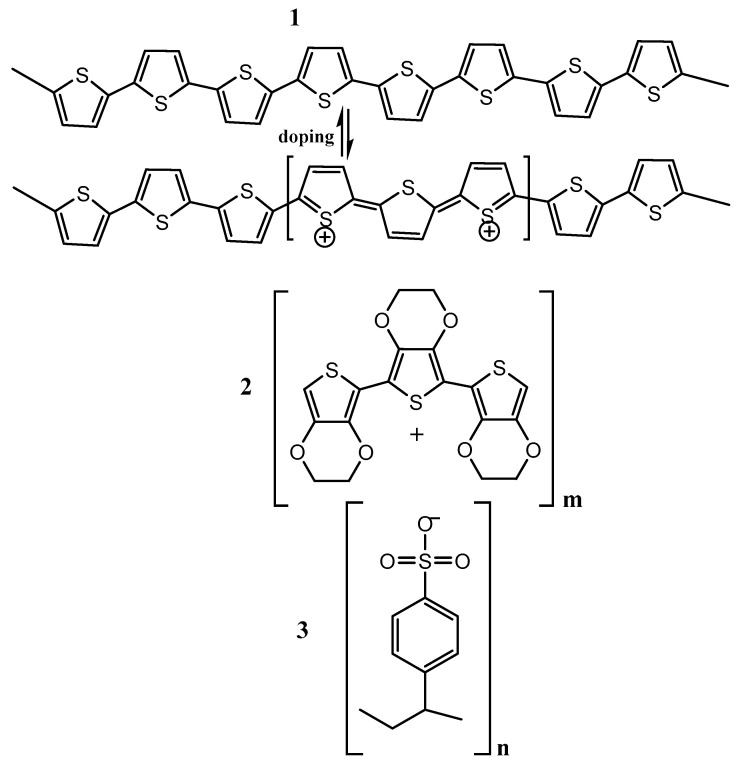
(**1**) polythiophene (PTP), (**2**) poly(3,4-ethylene dioxythiophene) (PEDOT), and (**3**) polystyrene sulfonate (PSS).

*Synthesis of Polythiophene (PTP) and Its Analogs*. With an electron-rich aromatic ring, thiophene is oxidized to form well-adhering polymeric films with high conductivity. The film thickness can be controlled by changing the polymerization time, and the structures of PTP and its analogs, as shown in Scheme 3. Of notice for bio-applications is a one-step enzyme entrapment by the EP process based on polythiophenes bearing oligo (ethylene glycol) spacers and porphyrin units [35]. 2,2′-bithiophene with lower oxidation potential, compared to thiophene, can be a good monomer. PTP and substituted PTP are electrochemically deposited as very adhesive thin films or as thick powdery deposits [36]. Poly(3,4-ethylene dioxythiophene)-polystyrene sulfonate (PEDOT: PSS) is a polymer mixture or macromolecular salt. PEDOT is a conjugated polymer with a positive charge, whereas PSS carries a negative charge due to the presence of deprotonated sulfonyl groups. There are four water-soluble polythiophene derivatives, which are synthesized via oxidative copolymerization by FeCl_3_ [37].

## 3. Fundamental Aspects of Electrochemistry and Biorecognition Elements

As the oldest analytical technique, electrochemistry offers a wide range of application possibilities by probing the charge transfer between an electrode and a liquid (electrolyte) or solid phase. In electrochemical sensing, an electrode serves as the transducer element to provide the electron for signal acquisition. This approach offers several advantages such as low cost, fast analysis, and ease of miniaturization. Associated with a biorecognition element, electrochemical sensors, known as biosensors, become specific for a target analyte, as exemplified by the selective detection of glucose using glucose oxidase or glucose dehydrogenase [38].

A typical electrochemical cell consists of three-electrodes: detecting (working), reference electrode, and counter (auxiliary). The use of the counter electrode, normally a large platinum wire with a small charge-transfer resistance, overcomes the potential drop due to the current flowing through the electrochemical cell. This configuration is needed for solutions with poor electrical conductivity. The measurement can be reduced to two-electrodes with a potentiostat that connects the working electrode with a non-polarizable reference electrode, e.g., Ag/AgCl (3 M KCl). Electrochemical techniques can be classified into five categories: potentiometry, amperometry, voltammetry, conductometry, and impedance spectroscopy. Potentiometry is based on the measurement of voltage between a detecting electrode and a reference electrode, e.g., pH electrodes and other ion-selective electrodes. Amperometry relies on a redox reaction at a detecting electrode in the presence of a target analyte, which results in the variation of an electrical signal. This method has been used widely in biosensor construction for a broad range of applications. Amperometry encompasses several techniques such as voltamperometry, cyclic voltammetry, differential pulse voltammetry, square wave voltammetry, linear voltammetry, etc. 

In brief, cyclic voltammetry is first performed to establish the oxidation/reduction potentials of analytes and assess whether the redox process is reversible, pseudo-reversible, or irreversible [39]. Voltamperometry (current response at a fixed potential) is then conducted to estimate the limit of detection (LOD), linearity, detection sensitivity, stability of the biorecognition element, and reproducibility for a given biosensor. Differential pulse or square wave voltammetry is useful for analyzing multiple analytes or endogenous reactive species in the same sample [40]. Conductometric relies on the measurement of conductivity or resistivity [41]. A capacitor changes its capacitance when exposed to the desired analyte, which binds to the capacitor’s surface. In impedance spectroscopy (EIS) or impedimetry, an alternative voltage (V_ac_) is applied with frequencies ranging from 10^−3^ to 10^5^ Hz. The resulting current at the same frequency (linear system) is measured. The resulting impedance (voltage/current) is represented on a Nyquist diagram, with the imaginary part as a function of the real part. Impedance spectroscopy has been used to probe cell behavior at an electrode surface in the presence of organic compounds, drugs, nanomaterials, etc., [42,43]. Commercial electrochemical workstations are well equipped with powerful software for conducting electrochemical experiments with different techniques. This subject has been well described in several textbooks [44] and review papers concerning electrochemical sensors/biosensors [45].

Biorecognition elements can be enzymes, DNA, peptides, aptamers, whole cells, etc. and the immobilization of such biomolecules on different types of electrodes has been available elsewhere [46]. Other special cases are the immobilization of glucose oxidase on an organic transistor using a polymer brush [47] and the hybridization of DNA on conducting polymer film-modified diamond [48]. Different biosensing formats have been attempted including the fabrication of an HRP (horseradish peroxidase)-based biosensor by thermal inkjet printing [49], a glucose sensor by a nanocomposite [50], and miniaturization technologies for biosensor design [51]. The potential of continuous measurement of analytes by biosensors in biological media is also reviewed [52].

## 4. Retention of Biorecognition Molecules by Conducting Polymers-Electropolymerized Polymers/Monomers

A single-step electrochemical immobilization method is used for the production of enzyme entrapped PANI thin films for glucose and urea sensors [53]. Both enzymes and PANI are deposited simultaneously from an aqueous solution of aniline containing optimized amounts of glucose oxidase and urease for the fabrication of two different biosensors. The chronopotentiometry curves for both the biosensors differ mainly in the total time taken for deposition. Other parameters, such as the type and enzyme concentration, have a negligible effect on the curve shape. The one-step immobilization technique has helped to produce functionalized films possessing an optimal working pH close to that of free enzymes to conserve their conformation and microenvironment. PPY, PEDOT: PSS, and polyaminobenzoic acid (PABA) are some common polymers used in biosensor fabrication [54,55,56,57]. A biosensor for 17β-estradiol detection is fabricated based on a benzothiadiazole derivative of EDOT [58]. The use of this polymer leads to the formation of a granular defect-free surface structure with a pore diameter of 1 μm. This porous structure allows enzyme molecules to anchor freely in the available space with no covalent bonding. The fabricated surface has a high enzyme loading capacity and easy regeneration capability where the sensor can be regenerated just by washing with deionized water. A new semiconducting polymer poly(9-nonyl-2,7-di(selenophen-2-yl)-9*H*-carbazole) is used in designing biosensors for norepinephrine determination [59]. Laccase is immobilized on the surface by π-π stacking involving the interaction of π-bonds in the enzyme structure with π-bonds of the aromatic backbone. The interaction stabilized the tertiary protein structure, thus improving its stability and activity. In addition to providing a surface for biomolecule immobilization, the presence of a small amount of selenium in the polymer has a positive effect on laccase catalysis.

EP has been proved as a simple and effective method to deposit conducting polymers on the conductive surface. The property of conducting polymers can be manipulated just by changing the composition of electrolytes used for polymerization. EP is performed to deposit zwitterionic PEDOT conducting films with antifouling properties on a conducting substrate. A mixture of thiophene-based compounds bearing phosphorylcholine (poly(2-methacryloyloxyethyl phosphorylcholine)), carboxybetaine (poly(carboxy betaine methacrylate)), and sulfobetaine (poly(sulfobetaine methacrylate) groups enhances the anti-biofouling properties [60]. The surfaces show resistance to protein and cell adhesion, which would help in improving the shelf life of the biosensors. An antifouling zwitterionic polypeptide modified with an additional linker of four residues is prepared and used for cancer biomarker BRCA1 (BReast CAncer gene) detection [61]. The composite shows strong resistance to non-specific protein adsorption and enhanced stability. Molecularly imprinted polymers (MIPs) based sensors are low cost and simple to fabricate. A typical MIP would include functional monomers, crosslinking reagents, and template molecules. As the polymer template is removed, a microcavity is formed for specifically binding target molecules with the same shape and structure as a key and lock [62]. The overuse of antibiotics is a major public health concern as they increase the chances of being antibiotic-resistant. It also harms the environment because of the pharmaceutical effluent mixing in water bodies. Amoxicillin (AMO), is one of the most popular types of aminopenicillin. It is detected by a hybrid film of (poly(methacrylamide-vinyltrimethoxysilane (VTMOS)-tetraethoxysilane (TEOS)/AMO) spin-coated on a surface plasmon resonance (SPR) gold sensor. The fabricated sensor detects the AMO down to 73 pM and differentiates among similar molecules, both in the buffer and in water [63]. Artemisinin is also a well-known and widely used drug against malaria, a deadly disease in tropical regions of the world. Ortho-phenylenediamine (*o*-PD) is electropolymerized in the presence of artemisinin on the gold wire. It provides highly stable and effective binding cavities for the target molecule with a detection limit of 0.01 μM [64]. However, it is still unclear how the interaction of the target molecule with functional monomers in the polymer can influence the microcavity formation. Some proteins can easily react with the polymer and change the tertiary and quaternary structure of the template, thus lowering the sensor selectivity.

For controlling the properties of CPs, different strategies have been used, and one of them is chirality. Chiral CPs with shown diverse structures enhance electronic properties and also help in redox reaction and pH switching [65]. They can help to understand bio-related processes due to their remarkable molecular recognition capability and distinctive self-assembling properties. The fabrication of chiral sensors is a promising field of research for electrochemists. A chiral supramolecular polymer consisting of alanine-based naphthalene diimides and Zn ions (AlaNDI-Zn) [66] exhibits multifunctional properties. Its photoconductive and chemo-resistive characteristics can be used in developing various sensors. It detects toxic hydrazine with a detection limit of 3.2 µM.

Hydrogels are polymers with high water retention capacity resembling biological tissues, which show great potential in biomedical applications [67,68]. Various target responsive hydrogels have been developed for molecular diagnostics, and wearable biosensors. A hydrogel-based localized surface plasmon resonance (LSPR) sensor is useful for detecting the biomarker in human tears. Silica gold nanoshells with poly (*N*-isopropyl acrylamide-*co*-methacrylic acid) (PNM) hydrogels can be used to detect lysozyme and lactoferrin. It shows potential as an affordable screening tool for chronic dry eye conditions [69]. A multiplexed biosensor based on PANI hydrogel is fabricated using an inkjet printer. The printing process consists of three steps with a multi-nozzle inkjet printer. The PANI hydrogel is printed, followed by the deposition of platinum nanoparticles, and enzymes. The multiplexed sensor can detect glucose, lactate, and triglycerides simultaneously with high selectivity in real-time [70]. A list of different polymers used for the electrochemical biosensors along with the method of biomolecule immobilization and other sensor properties are given in Table 1.

## 5. Conducting Polymer Composites

The properties of polymers can be improved by the addition of fillers and the formation of composites. The most used materials for polymer composite formation are CNTs, carbon nanofibers, graphene and its derivatives, other carbon materials, metal nanoparticles, and metal oxide nanoparticles. CNTs improve the electrical and thermo-mechanical properties of CPs thin films. In general, composites of polymers with both multiwalled CNTs (MWCNTs) and single-walled CNTs (SWCNTs) have been used for several analytes with enhanced sensitivity and stability due to increased and faster biomolecule to electrode charge transfer as well as improved biomolecule activity. Graphene is another carbon-based nanomaterial that has been used widely in electronic devices. A field effect transistor (FET) based on a composite of graphene and poly(3-aminobenzylamine-*co*-aniline) exhibits extremely sensitive detection of acetylcholine. The amine-functionalized polymer provides a suitable environment for enzyme immobilization and improves the pH sensitivity of the graphene-based FET owing to a wide pK_a_ distribution of polymer components [97]. An electrochemical comparison between GO/PEDOT and rGO/PEDOT composite reveals that GO enhances the capacitive behavior of the polymer film more than rGO [98]. Currently, porous carbon with a high conductivity and surface area has emerged as a new cost-effective material for screen printed electrodes used in biosensing devices. Its electrocatalytic properties can be further enhanced by doping carbon with heteroatoms like B (boron), N (nitrogen) or S (sulfur). The detection of neurotransmitters and lactic acid is feasible using the composites of N and S doped carbon with polymers like poly 3-((2,2′:5′,2″-terthiophen)-3′-yl)-5-aminobenzoic acid (pTTABA)) and 2, 2′:5′,5″-terthiophene-3′-p-benzoic acid (TBA) [99,100]. The core-shell nanocomposite of Pt-Pd with N-doped carbon shell acetylcholine biosensor is fabricated for organophosphate pesticide detection in “real-world” samples. The composites of porous carbon with polymers show better electrocatalytic properties and conductivity. Its detection limit for malathion, chlorpyrifos, and parathion is 7.9 × 10^−15^ M, 7.1 × 10^−14^ M, and 8.6 × 10^−15^ M, respectively [101]. Carbon dots (CDs) are the fluorescent nanosized carbon particles which have attracted much attention since their discovery. In comparison to other carbon materials, CDs are easy to synthesize, small, hydrophilic, and can be modified with various functional groups. CDs with fluorescent and photophysical properties are often mixed with polymers to enhance the electron transport and stimulate fast redox reactions. CDs are mixed with chitosan for the determination of glucose as the composite enhances the electron transfer and provides a biocompatible sensor surface. The CDs increased the conductivity and 3-aminobenzeneboronic acid as the monomer for the fabrication of MIP. The fabricated sensor exhibits a limit of detection (LOD) of 0.09 μM with excellent stability, and reproducibility [102]. Caffeic acid (3,4-dihydroxycinnamic) is a widely used drug for anti-inflammatory, leucopenia, and thrombocytopenia, and hemostasis. A CDs@MIPs based sensor detects this acid in human plasma with an LOD of 0.11 μM and a linear range of 0.5–200 μM [102].

Graphitic carbon nitride (gCN) is a synthetic polymer with an increase in interest in applications ranging from photoelectrochemistry, (photo)catalysis, and, biosensors [103]. Its reactive interface with an aromatic π-conjugated framework provides opportunities to control its structure and properties for a specific application. The gCN is incorporated with MIP to fabricate a self-powered biosensor based on photo-fuel cell, resulting in good detection selectivity for bisphenol A. The sensor is powered through the catalytic activity of Ni(OH)_2_ toward the oxidation of glucose [104]. An electroactive nanocomposite of Ru@Au core-shell nanoparticles, gCN nanotubes, and functionalized graphene quantum dots is used to detect phenylethanolamine A [103]. The fabricated sensor with an LOD of 2.0 × 10^−13^ M is tested on the urine sample with good selectivity for phenylethanolamine A. Gold nanoparticles (AuNPs) are the most used metal nanoparticles in biosensors. An interesting antifouling biosensing platform is constructed using AuNPs and PEDOT, where AuNPs acted as signal enhancers, whereas the polymer assisted in the antifouling behavior of the sensor surface [105]. The device is tested in biological media for CA15-3 detection, and it shows an elongated antifouling performance with only a negligible activity change during 30 days. Natural clay has been under research as a transducer surface modification agent due to its stability, porosity, high cation exchange capacity, low price, and widespread availability [106,107]. A glucose biosensor based on a platinum disk modified with PANI, chitosan, montmorillonite clay particles, and platinum nanoparticles shows extended stability up to two months with excellent stability and selectivity [108]. Thus, clay particles can be used to increase the stability of the polymer surface-modified electrode. Of interest is the fabrication of poly (3,4-ethylenedioxythiophene)-iridium oxide nanocomposite based tyrosinase biosensor for the dual detection of catechol (LOD = 0.017 µM) and azinphos methyl (LOD = 2.964 µM) [109]. Anziphos methyl is a broad spectrum organophosphate insecticide and the measurement is based on the inhibition of the activity of tyrosinase. Ferrocene-substituted 2,5-di(thienyl)pyrrole (SNS-Fc) can be electrochemically polymerized with 3,4-ethylenedioxythiophene (EDOT) to serve as an immobilization matrix of GOX by cross-linking in the presence of MWCNTs [110].

As illustrated in Table 2, conducting polymer-based composites are formed by blending or mixing CPs with other materials to achieve multifunctionality [144]. Resulting composites benefit from the synergistic effect of different components to exhibit multi-functionalized properties with enhanced mechanical performance and processability. The interfacial adhesion between the conducting polymer and other components affects the morphologies and electrochemical properties and morphologies of composite films, which can be attributed to novel composite properties. The presence of the amino group of aniline is needed for bioconjugation with the amino group or the carboxyl group of the biorecognition element by glutaraldehyde crosslinking or carbodiimide activation, respectively. Noble metal nanoclusters, e.g., platinum nanoclusters, can be integrated during oxidative polymerization of pyrrole to form a composite with enhanced electrochemical properties, mechanical performance, and processability [145]. The conducting polymer-based composite exhibits electrocatalysis for glucose oxidation with a linear range of 1–30 mM. However, the signal response is not provoked by common electroactive interferents such as ascorbic acid, uric acid, and 4-acetamidophenol in blood glucose analysis.

## 6. Polymers Conjugated Mediators for Direct Electron Transfer (DET)

As third generation biosensors are mainly based on DET from the enzyme–electrode surface, the polymer matrix can serve as an efficient surface for mediator attachment, thus preventing the leaching of mediator molecules and improving the electron transfer as well. Ferrocene and its derivatives are very popular redox mediators used in biosensor construction. They are very stable in both oxidized and reduced form, have excellent redox reversibility, and their redox capacity is not affected by the pH of the solution [110,140]. A highly sensitive reagentless glucose detection electrode is designed with copolymerization of ferrocene and functionalized PPY. The sensor has a detection limit of 0.43 μM with a sensitivity of 23.12 μA mM^−1^ cm^−2^ and can detect trace analytes [110]. The functionalization with the mediator shifts the working potential from 0.6 V to 0.4 V and prevents mediator leaching, thus increasing stability and limiting the interference in real samples. A biosensor surface is modified with ferrocene and PPY for bacterial detection with a LOD of 100 fM [140]. A sensitive microfluidic set-up coupled with a modified SPE is designed for neurotransmitter detection and able to detect the target analyte as low as 34 pM [100]. Titanium yellow, an aromatic anionic compound, can be used to enhance PPY electroactive properties. The polymeric film with sulfonate functional groups provides enhanced conductivity and stability compared to chlorine [146].

Evans blue acting as a mediator is attached to the polymer surface by covalent linkage. It acts as an electron mediator and aids in selectivity enhancement by attracting cationic species and repelling anionic counterparts due to the presence of negatively charged sulfonate groups. The device provides a rapid, low cost and effective method for neurotransmitter detection and avoids the use of specific labels. Viologen is a well-known redox molecule containing a 4,4′-dipyridinium group. It undergoes two-electron transfer processes and exhibits three redox states, namely V^2+^, V^+^, and V^0^, making it suitable for electrochemical applications. This property of viologen is used to modify carbazole monomer by electropolymerization, which exhibits enhanced thermal stability and a large electrochemical window [131,147]. The polymer-based electrode enhances the oxidative current in the detection of uric acid and ascorbic acid. However, the mediator modification does not always increase the sensor performance. Nickel hexacyanoferrate (NiHCF) is used as a mediator for layer by layer EP of PPY-PSSA and PPY-GOX or lactate oxidase. The sensor without a mediator has higher sensitivity of 0.94 nA/mM for glucose than the mediator modified sensor (0.32 nA/mM) [148]. A list of various mediator conjugated polymer platforms used in 2020 for biosensing is given in Table 3**.**

## 7. Non-Enzymatic Detection of Glucose

Enzymeless electrochemical determination of glucose has been a dream of many researchers to overcome the drawbacks of poor stability and the high cost of GOX. In this context, considerable attempts for non-enzymatic detection of glucose using CPs, particularly, PPY and PANI, have been noticed and, in most cases, metal or metal oxides are required to effectuate in electrocatalysis (Table 4). The CPs aid in increasing the stability and enhancing the electrochemical capacity of the electrodes. In contrast, biosensors are highly specific and less vulnerable to interference, but the working conditions and stability are still major drawbacks [132]. The measurements are performed in alkaline solution, which is not very attractive to patients with diabetes mellitus, and the linear range is very narrow, falls short of the required limit of 25–30 mM. Non-specific protein binding on metal particles and conducting polymers are expected to foul the detecting surface and affect the signal response. Attempts have been made to improve the conductivity and hence sensitivity using a combination of CPs, metal, or metal oxides with carbon nanotubes or graphene. There are still formidable challenges concerning the applicability of enzymeless detection of glucose using blood [155]. Novel materials offering better electrodes and protective films with antifouling properties should be investigated to yield mature technologies applicable to the commercialization of non-enzymatic sensors. Non-specific adsorption of proteins and biomolecules is always problematic when more hydrophobic materials are present on the electrode.

## 8. Trends and Future Possibilities

It has been a long journey since the discovery of CPs with a Nobel Prize awarded to three people: H. Shirakawa, A. G. MacDiarmid, and A. J. Heeger in 2000 [170]. Considerable attempts have been focused on the use of CPs as electrode materials in batteries [171]. There are still several cost and technical issues for the use of CPs in this domain, compared to optoelectronic devices [172]. Electrochemistry has advanced significantly from the pioneering concept and historical use of the mercury electrode by Heyrovsky [173]. A fundamental framework of direct electron transfer (DET) was elucidated and solidified by Marcus, another Nobel Prize winner in Chemistry [174] A search from Web of Science unravels 2500 publications related to the use of CPs for biosensing, starting with the use of two classical CPs, PANI and PPY. Other polymers have emerged and the PEDOT: PSS pair has also received attention, whereas several other CPs have not been attempted [172]. More publications related to CPs have also been supported by nanoparticles, carbon nanotubes, graphene, etc. to form polymer composites. Pertaining to the synthesis of CPs, the polymerization of carbon dots is worth noting [175]. Conducting polymers decorated with nanoparticles such as CuO, zinc doped CuO particles exhibit antimicrobial activities [176,177]. This feature might open the possibility for the development of implanted biosensors or enzymeless detection of glucose based on electrocatalysis of such metal oxide nanoparticles.

In principle, biosensors are versatile tools for monitoring of a variety of analytes with significant importance in healthcare, agriculture, environment, food, and biosecurity. The confinement of a recognition molecule on highly conductive electrodes is necessary for the development of sensitive and selective biosensing. Besides glucose monitoring or pregnancy test, the commercialization of other types of biosensors is tepid, including the one for cholesterol. Even glucose biosensors encounter tough competition with non-invasive glucose monitoring systems, equipped with smartphones for data processing, storage, and transmission. Non-specific protein adsorption is always problematic and challenging, which might impede the performance of conducting polymer biosensors. Most of such biosensors have been evaluated under controlled laboratory conditions, and clean samples are spiked with various known interfering molecules. Clinical samples might contain several unknown plausible interferents, which invoke a deficiency in both specificity and selectivity of target molecules. For medical applications, the practice is merely based on single-use, so the issue of simple adsorption on the electrode or covalent coupling of the sensing biomolecule to a conducting polymer becomes very trivial. For environmental monitoring, the degradation of CPs over time is likely unavoidable. Thus, intensive research is still needed on the stability improvement of the biosensors.

The role of existing CPs and the search for new ones need to be addressed from both commercial and academic viewpoints. Pyrrole and its derivatives possess all desirable features for the fabrication of biosensors and immunosensors. In brief, they are stable under ambient conditions and polymerized under natural and aqueous media for the enzyme entrapment process in one single step. Extensive research has been conducted with PPY for the development of chemical sensors, biosensors, DNA-based sensors, and immunosensors.

A similar situation is expected for PANI with an amino group for conjugation of recognition biomolecules as this polymer has been investigated rather extensively in biosensors construction. PANI and its derivative are well-known for their semi-flexibility and high conductivity to serve as immobilization matrices for biomolecules without compromising their biological activities. It can still serve as an immobilization matrix for bioconjugation; however, it must compete with other polymers and biopolymers, including Nafion, chitosan nanocrystal, starch, nylon, cellulose nanocrystal, etc. For long-term applications, pure CPs have several shortcomings such as decreased sensitivity, reduced selectivity, surface poisoning, and other agent intrusions on their pore structures. The issue of polymer aging should be investigated carefully as it might affect the polymer matrix disorder and permselectivity, resulting in biosensors with poor analytical performances. To a certain extent, CPs can be formulated with graphene, carbon nanotubes, and metallic nanoparticles. Polymer composites could play an important role in the development of wearable and flexible high-performance sensors/biosensors.

The demand for glucose monitoring will inevitably increase significantly as 450 million cases of diabetes could reach 700 million cases in 2045 [178]. The future trend of glucose detection will focus on the different bands of glucose in the electromagnetic spectrum with the aid of advanced mathematical and statistical algorithms to improve the accuracy of glucose estimation [179]. Doubtlessly, the appearance of non-invasive glucose is emerging, and it is just a matter of time before non-invasive monitoring of glucose with acceptable accuracy and specificity is achieved. It is also questionable if continuous monitoring of glucose is needed except for critical diabetes type 1. A comprehensive review of the performance of non-invasive, minimally invasive biosensing for glucose is available elsewhere. Accuracy, selectivity, and precision are always plausible caveats that need to be carefully investigated and solidified, a backdrop for future research endeavors. In emergency departments, clinical diagnosis of glucose, lactate, cholesterol, urea, creatine, and creatinine is performed. Thus, the trend is moving toward the development of integrated biosensors for multi-analytes detection. In this context, the stability of such integrated biosensors is more problematic since this requirement is dependent from one enzyme to another one, which creates significant obstacles to the practical applications. For successful commercialization, the enzymes must be stable for at least 6 months with unique detection selectivity. To date, glucose oxidase is the only enzyme that fulfills these tough requirements, and it is not surprising to see more publications with glucose detection as over 100,000 papers have been published.

The approach of using enzymeless sensing is questionable for any compound due to its lack of selectivity for the analysis of “real world” samples. One example is the detection of nitrite in food, an approved food preservative. Nitrite is a precursor of nitroamines, a suspect cancer agent, so its level is regulated at safety levels [180]. Among various methods, nitrite can be oxidized to nitrate by a gold electrode modified by AuNPs and *p*-aminothiophenol [181]. However, ascorbic acid, an endogenous electroactive species, is also detected to cause significant interference and must be removed by ascorbic acid oxidase if the measurement is based on simple amperometry. Differential pulse voltammetry and square wave voltammetry are more applicable in this situation, provided the potential difference between a target compound and interfering species is sufficient.

The detection of ethanol as an indicator of “driving under influence” is a common practice in many countries. Alcohol oxidase is available, and there have been several attempts with this enzyme for the detection of ethanol in liquid. However, vapor ethanol is well served by different breath analyzers, and this field has been very competitive and crowded with four different approaches: color change due to a chemical reaction, infrared spectroscopy, fuel cell technology, and combined infrared and fuel cell. There has been no significant progress in the respective biomolecule immobilization. Simple adsorption of biomolecules on insoluble supports is attributed to nonspecific hydrophobic interaction and hydrogen bonding. Entrapping biomolecules by polymers, e.g., Nafion is very effective to confine proteins on the electrode. However, bioconjugation is more commonly used to form stable covalent binding between biorecognition molecules with the support for repeated analysis. This technology is maturing, and the most used techniques are still glutaraldehyde and carbodiimide activation.

Based on the solid-phase synthesis, peptide chains with high specificity and versatility can be synthesized and custom-made to meet specific requirements. Compared to other biological materials, peptides are more stable and active for prolonged periods, compared with enzymes and antibodies. Peptides can also be designed to have specific three-dimensional structures like proteins to improve biorecognition capabilities like antibodies and enzymes. The future development of peptide-based biosensors is expected to grow significantly. Considerable demands are also expected for the detection of food and waterborne parasites. *Cryptosporidium*, also known as Crypto (diarrhea linked to water) and *Giardia* (diarrheal illness is known as giardiasis caused by a parasite) are two important targets. Besides human infection, many species of *Cryptosporidium* infect animals, i.e., the application of biosensors in veterinary medicine could be enormous if it is cost and performance effective, compared to immunoassays. Of importance, is the development of advancing peptide-based biorecognition elements for biosensors using in-silico evolution [182].

Recent progress in the synthesis of porous carbon materials deserves a brief discussion here because the use of these materials in biosensing is very limited. Porous carbon materials have three types of pore sizes: microporous (below 2 nm), mesoporous (>2 nm and <50 nm), and macroporous (above 50 nm) and can be synthesized by several different methods [183]. Porous carbon (PC) has several distinct features such as high surface areas (over 2000 m^2^ g^−1^), tunable surface chemistry, and short diffusion pathway for rapid mass transfer. Albeit PC has been used extensively in electrocatalysis, energy storage, and capacitors, its application in biosensors is very limited [184,185] PC can simply form a nanocomposite with Prussian Blue (PB) and serve as an efficient immobilization matrix for GOX toward the development of a biosensor for glucose detection [186]. As electrodes for biosensing, uniform porous carbon materials with highly graphitic structures are needed and the synthesis of such materials has been very challenging. Macroporous graphite felt and porous graphitized carbon monolith are two materials that could be used to fabricate biosensors [187] or high-performance microbial cell anodes [188]. The use of CPs with porous carbon remains to be explored, a backdrop for future research endeavors.

## 9. Conclusions

Both PANI, PPY, two classical conducting polymers, and their derivatives have been exploited extensively towards the development of different biosensing platforms. Conducting polymer-based composites are formed by blending or mixing CPs with other materials, including metal oxide nanoparticles to achieve multifunctionality. Resulting composites benefit from the synergistic effect of different components to exhibit multi-functionalized properties with enhanced mechanical performance and processability. The interfacial adhesion between the conducting polymer and other components is attributed to composites properties, structures, and morphologies. Noble metal nanoclusters, e.g., platinum nanoclusters, can be integrated during oxidative polymerization of different monomers to form a composite with enhanced electrochemical properties, mechanical performance, and processability. Detection of immunoglobulins such as IgM, IgG, and IgA in response to infectious diseases, will receive considerable attention. Synthesized peptides and recombinant antigens and proteins of viruses and pathogens can be synthesized and used as biorecognition elements. CPs and their composites are anticipated to serve as excellent immobilization matrices for such biomolecules. Emerging porous carbon and carbon felt have potential applications in both chemosensing and biosensing platforms due to their high surface areas and ease of synthesis.

## Figures and Tables

**Table 1:** The use of polymers to retain the recognition molecules or modification of the electrode surface.

Polymer (s)	Analyte	Biomolecule Immobilized	Immobilization Method	LOD	LDR	Ref.
PANI	Glucose (G) and urea (U)	GOX/URE	Entrapment	-	0.1 mM (G) 10^−5^–0.1 M (U)	[53]
PPY-pTS	Hypoxanthine	XOX, URI	Entrapment	5 µM	5 µM–5 mM	[54]
PPY	Interleukin-10	AIA	Covalent	0.347 pg/mL	1–10 ng/L	[55]
NDSC	Norepinephrine	Laccase	Adsorption	240 nM	0.1–200 µM	[59]
PEDOT	BRCA1	DNA	Covalent	0.0034 pM	0.01 pM to 1 nM	[61]
PEDOT-PEDOT-COOH	Lactate	LDH	Covalent	20.26 μA mM^−1^ cm^−1^ (sensitivity)	20–960 μM	[71]
PEDOT/PSS hydrogels	H_2_O_2_	HRP	Nafion entrapment	0.94 µM 10 µM	0.0088 to 0.15 mM 0.05–0.25 mM	[72]
PPY/PSS	Glucose	GOX	Entrapment	-	0.1 nM–1 mM	[73]
DMAPM, VPBA, EGDM, DA	Dopamine	Imprint	Entrapment	96 nM	40 nM–20 μM	[74]
MAA, EDGM, fluorescein-o-acrylate	Doxycycline	Imprint	Entrapment	117 nM	0.2 to 6 µM	[75]
Toluidine blue	CA 15–3	Imprint	Entrapment	0.10 U/ mL	0.10 U/mL to 100 U/mL	[76]
PDA	TNT	Imprint	Entrapment	15.4 nM, 14.8 nM	0–289 nM	[77]
o-DB, o-PD	Chlorpyrifos	Aptamer imprint	Entrapment	0.35 fM	0.001 to 0.4 pM	[78]
Macroporous PANI, PSS	AFP	AFP Ab	Covalent	3.7 fg/mL	0.01 to 1000 pg/mL	[79]
CuPT-PPy/NIPAAm-AM	Melanoma	CRP	Covalent	7.85 × 10^−19^ M	10^−20^ to 10^−6^ M	[80]
NiPAAm, AAm, MBAAm	BSA	Imprint	Entrapment	0.012 μM	0.02 μM to 10 μM	[81]
FcMMA, 4-VP, and EDMA	Bisphenol A	Imprint	Entrapment	13 ng/L	0–410 ng/L	[82]
PEDOT	NADH		Physical adsorption	5.3 μM	20–240 μM	[83]
PLL	DNAhybridization	DNA	Covalent	2.25 × 10^−14^ M	1.0 × 10^−13^ to 1.0 × 10^−6^ M	[84]
PPY	CEA	Imprint	Entrapment	0.13 pg/mL	0.125–12.25 pg/mL	[85]
PCPy	Glucose	GOX	Entrapment	0.039 mM	0.1–15.0 mM	[86]
PSBEDOT	Glucose	GOX	Entrapment	-	1 mM to 20 mM	[87]
BTP	Glucose	GOX	Entrapment	0.034 mM	0.034–1.0 mM	[88]
PPY	Glucose	GOX of *A. niger*	Coating the cell	0.005 mM	0.01–0.05 mM	[89]
PPY	Catechol	POX (*Trametes pubescens*)	Encapsulation	1.8 μM	1–60/70 μM	[90]
p-toluene-sulfonic acid doped PPY	Glucose	GOX	Physical adsorption	0.25 mM is lowest tested	0.25–1.5 mM	[91]
BOTT	Catechol	Laccase	Entrapment	0.38 μM	0.5–25 μM	[92]
PEDOT(BSA)	Glucose	GOX	Physical adsorption	0.416 mM is tested	0.416 to 50 mM (LSV)	[93]
DPDAA	Glucose	*G. oxydans* and GOX	Glutaraldehyde cross-linking Cells:	0.022 mM (GOX) 0.081 mM (whole cell)	0.045–50.0 mM (GOX) and 0.19–50.0 mM (whole cell)	[94]
PANI	*E. coli*	Anti-*E. coli* antibody	Covalent	10 CFU/mL	10^1^–10^6^ CFU/mL	[95]
Macroporous PPY-pyrrole	Urea	Urease	Entrapment	2.57 mM	1.67–8.32 mM	[96]

*Polymers*: AAm = acrylamide, BOTT = thienothiophene-benzoxadiazole-alt-benzodithiophene, BTP = 5-amino-N1,N3-bis (2,5-di(thiophen-2-yl)-1*H*-pyrrol-1-yl) isophthalamide, DA = 3-hydroxytyramine hydrochloride, DMAPM = *N*-[3-(dimethylamino)propyl]methacrylamide, DPDAA = 10-(4*H*-dithiyeno [3,2-b:2′,3′-d]pyroll-4-il)decan-1-amine, EGDM = ethylene glycol dimethacrylate, FcMMA= Ferrocenyl methylmethacrylate, MAA = methacrylic acid, MBAAm = methylene-*N*,*N*-bis(acrylamide), NDSC = 9-nonyl-2,7-di(selenophen-2-yl)-9*H*-carbazole, NiPAAm = *N*-isopropylacrylamide, o-DB = o-Dihydroxybenzene, PANI = polyaniline, PBETB: poly(4,7-bis(5-(3,4-ethylenedioxythiophene)thiophen-2-yl)benzothiadiazole), PEDOT = poly(3,4-ethylenedioxythio phene), pTS = para toluene sulphonate, PLL = Poly-L-lysine, PPy = poly pyrrole, PSBEDOT = poly(sulfobetaine-3,4-ethylenedioxythiophene), PSS = poly styrene sulphonate, VPBA = 4-vinylphenylboronic acid, 4-VP = 4-vinylpyridine. *Biorecognition biomolecules*: AFP = alpha-fetoprotein, AIA = Anti-Interleukein antibody, BSA = bovine serum albumin, CA 15-3 = Carbohydrate Antigen 15-3, CEA = Carcinogenic embryonic antigen, CRP = C-reactive protein, GOX = glucose oxidase, HRP = horseradish dehydrogenase, LDH = lactate dehydrogenase, POX = polyphenol oxidase, TnT = Cardiac Troponin T, URA = urease, URI = uricase, XOX = xanthine oxidase.

**Table 2:** Polymer nanocomposites containing carbon nanotubes (CNTs), graphene, and nanoparticles in biosensors.

Nanocomposite	Target Analyte	LOD	LDR	Ref.
PEDOT: PSS/Fe_2_O_3_	Carcinoembryonic antigen	-	4–25 ng/mL	[56]
PABA and Graphene	Acetylcholine	5 to 1000 μM	2.3 μM	[97]
PTAB and N, S doped porous carbon	Lactic acid	0.5 μM and 4.0 mM	112 nM	[99]
PEDOT and AuNPs	CA15-3	0.001−1000 U/mL	0.32 mU/ mL	[105]
MnO_2_/PAA-GG	H_2_O_2_	10 μM	0.05 mM−50 mM	[111]
PEG/graphene/aptamer	IgG	47 pM	50 pM–250 nM	[112]
PQQ-GDH/PTh/MWCNT	Glucose	1 μM	1 μM to 2 mM	[113]
PANI/MWCNTs/Starch	Cholesterol	0.01 mM	0.032 to 5 mM	[114]
GOX-PtNPs-PEDOT-MS	Glucose	1.55 µM	0.1–10 mM	[115]
CeO_2_-NRs/Ppy-NPs ssDNA probe (covalent)	DNA from *Salmonella*	0.29 µM	1.0 nM−1 µM	[116]
PIn-5-COOH/MWCNTs-COOH	α-fetoprotein	0.33 pg/mL	0.001–100 ng/mL	[117]
poly(pyrrole-3-carboxylic acid)/GO with GOx	Glucose (ITO coated glass)	0.05 mM	1–20 mM	[118]
PAnNFs-P(An-co-PoPD)]	Glucose (GOX)	Minimal glucose conc. tested is 5 µM	Saturation at 0.45–0.6 mM	[119]
PDA-MWCNTs-PB	Cholesterol (Ch) (Ch oxidase/SPE)	1.5 µM	Linear up to 0.4 mM	[120]
PEG-PPY nanowires	DNA probe for miRNA	0.033 pM	0.10 pM−1.0 nM	[121]
rGO-PEDOT:PSS	GOX fpr glucose	86.8 µM	Detection range: 100 µM to 600 µM	[122]
PPY-Ag/ZnO on pencil graphite electrode	Xanthine (X) (X oxidase)	0.07 µM	0.06–0.6 µM	[123]
P(SNS-An-co-EDOT)	Glucose (GOX)	1.9 µM	0.01–5.0 mM	[124]
PPY-CNTs/(SrCuO_2_)	2,4-dichlorophenol (laccase-graphite electrode)	0.18	1–50 µM	[125]
PPY bearing the redox dendrimer PAMAM G2	autocrine motility factor (cytokine)-cancer biomarker	43 fM	1 pM−1 µM	[126]
PANI/PPY/MWCNTs-COOH	H_2_O_2_ (Cytochrome C)	0.1 µM	1–370 µM	[127]
ePDA	AA (Laccase)	The lowest AA tested is 7 µM (CV mode)	Sensitivity = 342 mA/Mcm^2^ with a linearity of 1–25 µM	[128]
Pt/rGO/P3ABA	-Glucose (GOX) -Cholesterol (ChOx)	44.3 µM 40.5 µM	0.25–6.00 mM 0.25–4.00 mM	[129]
PtNPs, MWCNTs-COOH–PEDOT:PSS	Glutamate Glutamate Oxidase	0.5 µM at 0.5 V 0.5 µM at −0.2 V	1 µM and 800 µM 10 µM and 600 µM	[130]
Cu-BTC-PANI on ITO	*E. coli* (anti-*E.coli* antibody)	*E. coli* (2 cfu/mL)	Linearity: 2–2 × 10^8^ cfu/mL	[131]
PBA-pTBA	Glucose (GOX)	0.29 µM	1 µM–30 mM	[132]
PDA	ABTS (Laccase)	0.29 µM	1–150 µM	[133]
PANI/rGO/Au NPs	Glucose GOX	64 µM	1–10 µM	[134]
PAB/DNA-functionalized CNT/Nitrogen doped graphene	Dopamine	14 nM	0.02–1 µM	[135]
PANI/Au NPs	Prostate-specific antigen Peptides	0.085 pg/ mL	0.1 pg/mL to 100 ng/mL	[136]
PAA/VS-PANI/GPL-FePc-conducting hydrogel	Glucose GOX	6.4 µM	1–20 mM	[137]
MPNFs of SnO_2_/PANI	Glucose GOx/HRP	1.8 µM	5–100 µM	[138]
PANI/Polyacrylamide	H_2_O_2_	2.9 nM	0.01–50 μM	[139]
PPY	*M. tuberculosis*	0.36 aM	0.1 aM to 100 fM	[140]
Carbon black/PVDF/SPGMA	IL-8	3.3 fg/mL	0.01–3 pg/mL	[141]
AChE/HCS@PANI	malathion	1.0 ng/mL to 10 μg/mL	0.16 ng/mL	[142]
GO/AAM/MAA/MMA/NVP	Zika virus	10^−3^ to 10^2^ PFU/mL	2 × 10^−2^ PFU/mL	[143]

AA = 2,2′-azino-bis(3-ethylbenzothiazoline-6-sulfonic acid, ABTS = 2,2′-azino-bis-(3-ethylbenzothiazoline-6-sulphonic acid), AChE = acetylcholine esteras, BTC = 1,3,5-benzenetricarboxylic acid, CMA = 4-aminophenylacetic acid, CS = chitosan, PABA = poly(3-aminobenzylamine-co-aniline), ePDA = a co-polymer enriched by chains of dopamine in the open form in detriment of indole moieties, GPL-FePc = iron phthalocyanine functionalised graphene nanoplatelets, HCS = hollow carbon spheres, HRP = horse radish peroxidase, MMT = montmorillonite, MPNFs = Polymerized multiporous nanofiber, P(An-co-PoPD)] = poly(aniline-co-*N*-phenyl-o-phenylenediamine) nanoflakes, P(SNS-An-co-EDOT) = poly(benzenamine-2,5-di(thienyl)pyrrole-co-3,4-ethylenedioxythiophene, P3ABA = poly(3-aminobenzoic acid, PAA = polyacrylic acid, PAB = poly anilineboronic acid, PAMAM G2 = *N*-(5-phosphate-D-arabinoyl)-2-aminoethanamine, PAnNFs= Polyaniline nanofibers, PB = Prussian Blue, PBA = poly-3′(benzoic acid), PDA = polydopamine, PDTP = poly 2,5-di(thienyl)pyrroles, PEDOT = poly(3,4-ethylenedioxythiophene), PEG = polyethylene glycol, PIn-5-COOH = poly(indole-5-carboxylic acid), PPY = poly pyrrole, PQQ-GDH = pyrroloquinoline quinone dependent glucose dehydrogenase, PTAB = polymer (poly 3-(((2,2′:5′,2″-terthiophen)-3′-yl)-5-aminobenzoic acid, pTBA = poly(2,2′:5′,2′-terthiophene), PTh = polythiophene, PVDF= polyvinylidene fluoride, SPGMA = star poly(glycidyl methacrylate), TBA = 5″-terthiophene-3′-p-benzoic acid, VS-PANI = inyl substituted polyaniline.

**Table 3:** Biosensors consisting of mediator conjugated polymers for direct electron transfer.

Mediator-Polymer	Target Analyte/ Sensor Platform	LOD	LDR	Ref.
Evans blue-poly-TBA/S,N doped carbon	Neurotransmitters (Amp)	0.034 (±0.005) nM 0.044 (±0.004) nM	0.05−130 nM	[100]
Ferrocene-PPY/MWCNT	Glucose (Amp)	2.2–0.43 μM	0.43 μM	[110]
Ferrocene-PPY	*M. tuberculosis* (SWV)	1 aM to 100 fM	0.36 aM	[140]
Polymethylene blue-PANI/MWCNT	Cardiac troponin T (DPV)	0.10–8.0 pg/mL	0.040 pg/mL	[149]
Ferrocene-PAA hydrogel	miR-21 (DPV)	10 nM to 50 μM	5 nM	[150]
BG-PAA	Glucose (Amp)	0.25–5.0 mM 0.1–1.75 mM	0.055 mM	[151]
FcAPS/GCE	H_2_O_2_(Amp/Fluors)	10 μM to 10 mM	2.07 μM	[152]
FPS/MWCNT-PDDA	Acetaminophen (DPV)	3 to 1100 μM	0.6 μM	[153]
PEDOT/AuNPs-MWCNT-COOH	Catechol Laccase (DPV)	0.11 and 12.26 μM	Two linear ranges: 0.1–0.5 and 11.99–94.11 M	[154]

Amp = Amperometry, BG = Brilliant Green, FcAPS = ferrocene carboxylic acid (FcA) cross-linked amino-polyethersulfone film, Fluors = Fluorescence, FPS = ferrocene polymer nanospheres, PAA = polyacrylamide, PANI = polyaniline, PDDA = Poly diallyldimethylammonium chloride, DPV = differential pulse voltammetry, PPY = poly pyrrole, TBA = 5″-terthiophene-3′-p-benzoic acid.

**Table 4:** Non-enzymatic glucose detection with conducting polymers (CPs) matrices.

CPs	Metal Particles	Analytical Performance	Ref.
Polybithiophene	Pd microparticles	Linearity: 0.04 to 0.4 mM LOD: 7 µM (under alkaline conditions)	[156]
Keggin Polyoxometalate/MWCNTs	Mixed-valence Co(III) and Co(II) structures	Linearity: 0.1 mM to 10.0 mM LOD: 1.21 µM (in 0.1 M NaOH)	[157]
PPY nanowire	A nickel-cobalt-sulfide nanosheet	Two linear ranges: 2 μM to 140 μM 0.14 mM to 2 mM LOD: 0.82 µM (in 0.1 M NaOH)	[158]
Benzoic acid-functionalized poly(terthiophene)	AuNPs	Potentiometric response (in saliva): 0.32 µM–1 mM, LOD = 0.19 μM.	[159]
Poly (*o*-phenylenediamide)	Ag-NPs	Linearity: 0.15 to 13 mM LOD: 12 μM Detecting blood glucose, PBS pH 7	[160]
Poly(thieno[3–4-b]-1,4-dioxin-2-methanol)	Not required	Linearity: 1–9 mM In 0.1 M PBS	[161]
PANI nanofiber	NiO/CuO NPs	Linearity: 20 to 2500 μM LOD: 2 μM (in 0.1 M NaOH)	[162]
PPY	ZnFe_2_O_4_ magnetic nanoparticles	Linear up to 8 mM LOD: 0.1 mM, in).1 M NaOH	[163]
PPY nanowires	CuO-Cu_2_O NPs	Linear up to 8 mM LOD: 6.2 µM (in 50 mM NaOH)	[164]
Poly(3-octylthiophene)	Au NPs	Potentiometric, Linear: 5–30 mM, LOD: 0.2 mM, in boric acid-borate buffer, pH 9 plus 100 mM NaCl	[165]
PPY nanowire/nickel foam substrate	NiCo_2_O_4_	LOD = 0.22 μM, linearity: 0.001–20 mM (in 0.1 M NaOH)	[166]
Conductive Polyaniline Nanosheets (CPANINS)		LOD = 0.043 μM, Linearity: 1–1000 μM (in 0.1 M H_2_SO_4_)	[167]
PANI/MWCNTs	ZnO	LOD: 0.1 mM, two linear regions: 0.1 to 1 mM and 1 to 6 mM (10 mM NaOH)	[168]
PANI	NiO	LOD: 0.06 μM, linearity: 1–3000 μM	[169]

AuNPs = gold nanoparticles, AgNPs = silver nanoparticles, PANI = polyaniline, PPY = polypyrrole, MWCNT = multiwalled carbon nanotubes.
